# Seamless Positioning and Navigation by Using Geo-Referenced Images and Multi-Sensor Data

**DOI:** 10.3390/s130709047

**Published:** 2013-07-12

**Authors:** Xun Li, Jinling Wang, Tao Li

**Affiliations:** School of Civil and Environmental Engineering, University of New South Wales, Sydney 2052, NSW, Australia; E-Mails: jinling.wang@unsw.edu.au (J.W.); tao.li@student.unsw.edu.au (T.L.)

**Keywords:** positioning, GPS, geo-reference, image, vision, digital compass

## Abstract

Ubiquitous positioning is considered to be a highly demanding application for today's Location-Based Services (LBS). While satellite-based navigation has achieved great advances in the past few decades, positioning and navigation in indoor scenarios and deep urban areas has remained a challenging topic of substantial research interest. Various strategies have been adopted to fill this gap, within which vision-based methods have attracted growing attention due to the widespread use of cameras on mobile devices. However, current vision-based methods using image processing have yet to revealed their full potential for navigation applications and are insufficient in many aspects. Therefore in this paper, we present a hybrid image-based positioning system that is intended to provide seamless position solution in six degrees of freedom (6DoF) for location-based services in both outdoor and indoor environments. It mainly uses visual sensor input to match with geo-referenced images for image-based positioning resolution, and also takes advantage of multiple onboard sensors, including the built-in GPS receiver and digital compass to assist visual methods. Experiments demonstrate that such a system can greatly improve the position accuracy for areas where the GPS signal is negatively affected (such as in urban canyons), and it also provides excellent position accuracy for indoor environments.

## 1. Introduction

Ubiquitous positioning is considered to be the main goal for today's Location-Based Services (LBS). While satellite-based navigation has achieved great advances in the past few decades and has been applied to both military and civilian applications in a mature manner, positioning and navigation in GPS-challenged areas has remained a largely unsolved problem and thus is currently receiving growing attention. The availability of rich imagery of large parts of the Earth's surface under many different viewing conditions presents great potential, both in computer vision research and for practical applications [1]. We believe it can also bring enormous opportunities to the field of navigation and location-based services. Images associated with vision sensors have been researched for positioning and navigation purposes since early in the last century. They are superior to many other techniques because they can operate both indoors and outdoors. In recent years low cost built-in sensors on mobile devices (e.g., smartphones), especially high resolution cameras, have placed greater demand for a breakthroughs in their applications for location-based services (LBS).

The main function of vision-based navigation is to determine the position and possibly the orientation of the imaging sensor, so as to recover the user's (or platform) position which is assumed to have a known position and orientation relative to the sensor [2]. However, the significant difference between outdoor and indoor environments has divided the early stages of this research topic into two different groups. The traditional approach for outdoor vision-based positioning is to match the real time query image with reference images in a database. Whenever a match is found, the position information of this reference image is transferred to the query image and used as the user position. This is essentially an object-recognition and image retrieval problem. A great variety of work has been done to address the location recognition aspect by using different image matching techniques (e.g., [3,4]). A further improvement is to calculate the relative position between the query view and the identified reference view to obtain more accurate position estimation. In 2006 Zhang and Kosecka [5] first used a wide-baseline matching technique based on SIFT features to select the closest views in the database, then the location of the query view was obtained by triangulation. In [6] the orientation of the sensor was also estimated since the pose of the query view is obtained from plane-to-plane transformation. A building façade was used as dominant plane. On the other hand, indoor visual navigation has been considered a quite different field. Related research has mainly focused on robotic visual SLAM (e.g., [7]), and significantly different methodologies like structure from motion and stereo viewing [8] are adopted, which are not suitable/attainable to be extended to LBS for common users. The major reason for it lies in that indoor and outdoor environments create different scenarios, requirements, and sometimes contradictory conditions to the visual system. In terms of size indoor environments are limited to certain buildings while outdoor positioning requires regional or even global coverage. In terms of accuracy indoor positioning obviously poses a greater challenge. In terms of sensors used to assist vision sensors, satellite-based navigation system can only cover outdoors and WiFi is more likely to be used indoors. In terms of vision-based algorithms and methods, greater diversity can be found. For instance, the use of stereo vision to extract depth information is more suitable for indoors since the range for depth detection is limited by the baseline. The visual features are normally different: in outdoor environments artificial landmarks (e.g., buildings, road signs, *etc.*) feature primarily the edges and corner points, while for indoor environment features are richer in the shapes and textures. However, despite all these factors, vision is by its nature capable of working in a complementary manner to satellite-based technology. Therefore, it is high time that a consistent framework of image-based visual navigation technology to be developed, which is capable of filling the gap in satellite-based system deficiencies, providing coverage from outdoors, urban canyons to indoor environments.

Seamless vision-based positioning covering both indoor and dense urban environments has therefore attracted growing attention nowadays, especially for the research of navigation system that can be used on mobile platforms. A common approach is to use a visual device as the main or complementary sensor that collaborates with other on-board sensors. In [9] the authors present an algorithm for estimating a pedestrian's location in an urban environment, and include data from GPS, inertial sensors, probability maps and a stereo camera. In [10] the authors utilize GNSS and map-matching for outdoor positioning; multiple sensors as well as signals of opportunity are adopted for the indoor environment. From the perspective of vision-based navigation, the major difference between these two approaches lies in that the former uses a stereo camera to directly extract depth information, while the second is based on a single camera and query image matching. Following these two main streams, many vision-related navigation solutions designed for GPS-degraded environment can be found (e.g., [11–15]).

In this paper, we present a hybrid image-based positioning system that intends to provide a seamless indoor/outdoor positioning solution. The system is designed to be applied to mobile platforms such as smartphones and moving vehicles. It mainly uses visual input to match with geo-referenced images for positioning resolution, and also takes advantage of multiple onboard sensors, including a GPS receiver and a digital compass to assist visual methods in various aspects. The major contribution that distinguish this paper from other approaches in the literature lies in that the geo-referenced images function as a 3D map, which enables the position information to be obtained in six degrees of freedom (6DoF, namely sensor position and orientation) based on a single image. The function model with its stochastic model of the system have been developed and tested with real datasets.

The paper is structured as follows: in the next section, the methodology of the system is described; then outdoor positioning and indoor positioning will be discussed, respectively, in greater detail; we present the experiment results in Section 5, while in the last section the concluding remarks are presented.

## 2. Methodology

Positioning is an essential component in navigation systems. It mainly functions in two different ways: absolute self-localization (e.g., GPS) and dead-reckoning (e.g., inertial navigation system). In this paper, we propose a hybrid image-based navigation system that is capable of self-localization in both outdoor and indoor environments. It requires a mapping process where images of the navigation environment are captured and geo-referenced. The main improvements in this work are to geo-reference image feature points and use these features as 3D natural landmarks for positioning and navigation. By matching the real time query image with pre-stored geo-referenced images, the 3D landmarks represented by feature points are recognized and geo-information can be transferred from reference image to query image through these common feature points. Final positioning is based on photogrammetric space resection. To strengthen the robustness of the system, the main sensor camera has been calibrated for any biases. The inherent error such as mismatches and any error from the input go through a multi-level outlier detection process, including RANSAC, data snooping algorithms. More details can be found in our previous work [16].

One essential element of the system is image matching. The state of the art image matching algorithms usually consist of two parts: detector and descriptor. They first detect points of interest in the images being matched and select a region around it, then they associate an invariant descriptor or feature to each region. Correspondences may thus be established by matching the descriptors [17]. To suit the scenario of the system, we have adopted various image processing techniques: Harris corner detector [18] and SIFT [19] for feature-based image matching, and RANSAC [20] as well as area-based matching method for the detection of mismatches.

### 2.1. Image Geo-Referencing and Mapping

**Figure 1.**
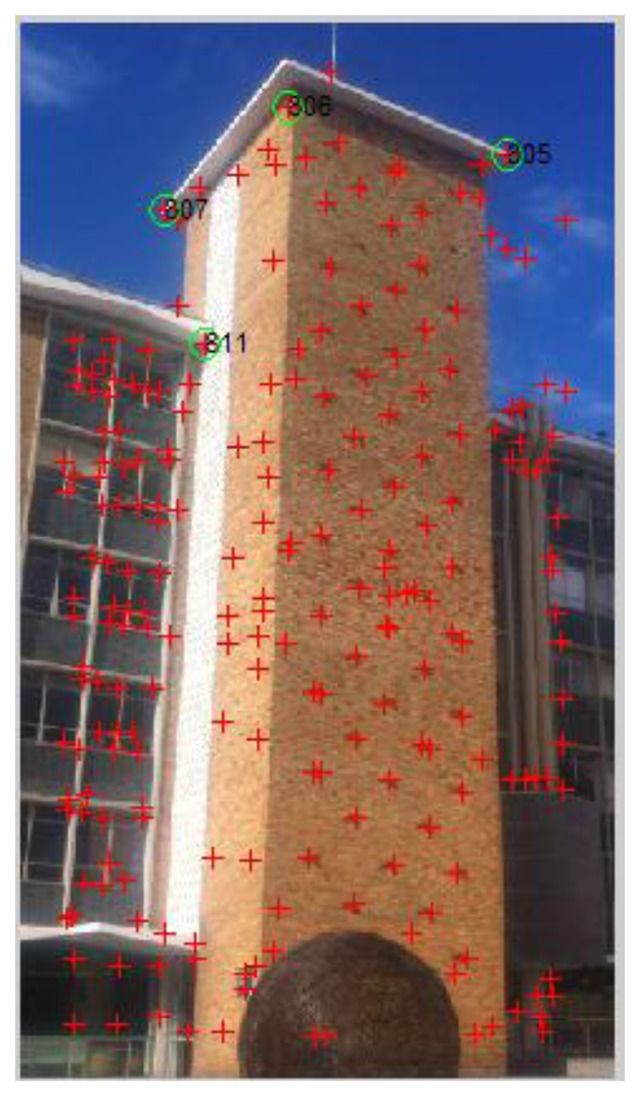
Reference image No. 15: Corner points have been extracted by Harris corner detector and shown with red crosses; 4 geo-located corner points have been identified and shown in green circle.

During the mapping stage, image feature geo-referencing is the core process. Different types of image features are used to cater different scenarios. In outdoor urban environments, artificial landmarks (e.g., buildings, road signs) feature primarily the edges and corner points. Therefore they are first surveyed in the field with derived Map Grid of Australia (MGA) coordinates. Then images are collected and the Harris corner detector is used to exact the corner features from these images. Among the big number of features points extracted, the ones that have been surveyed are identified from the feature list and associated with images with both 2D image pixel coordinates and 3D surveyed coordinates been recorded (e.g., Figure 1 and Table 1).

For landmarks that have a certain volume in space, such as buildings, images of façades are geo-referenced. Each building is described by the contour information (corner coordinates and edges they intersect) and the geo-referenced images, including both the outdoor façades and the indoor environments.

**Figure 2.**
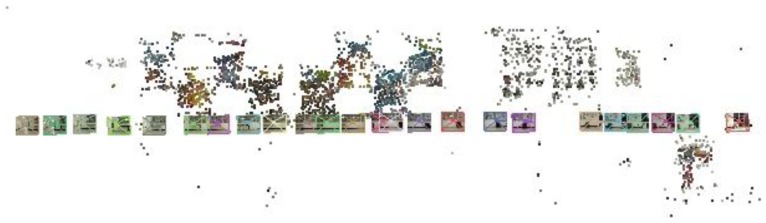
Visualization of SIFT features for geo-referencing (point cloud) produced by Wu's VisualSfM software from the reference images, which are shown by square patches.

**Figure 3.**
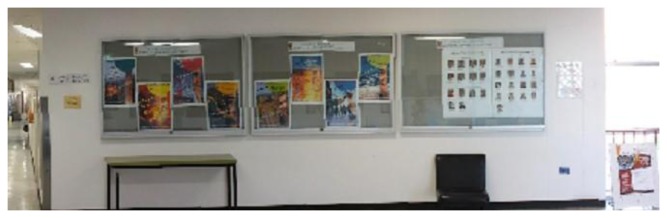
Panorama view of the mapped area in Figure 2.

For indoor positioning and navigation, higher accuracy is required. Additionally, the texture of the indoor environment, which may contain furniture, walls, posters and curtains instead of corner features is different from the outdoors. Therefore, we have introduced SIFT feature points for geo-referencing in the indoor mapping stage. This method increases the density of geo-referenced feature points and does not rely on corner features. For better illustration, Wu's VisualSfM software [21] is used to visualize the features (in Figures 2 and 3), but this software has not been used in data processing.

**Figure 4.**
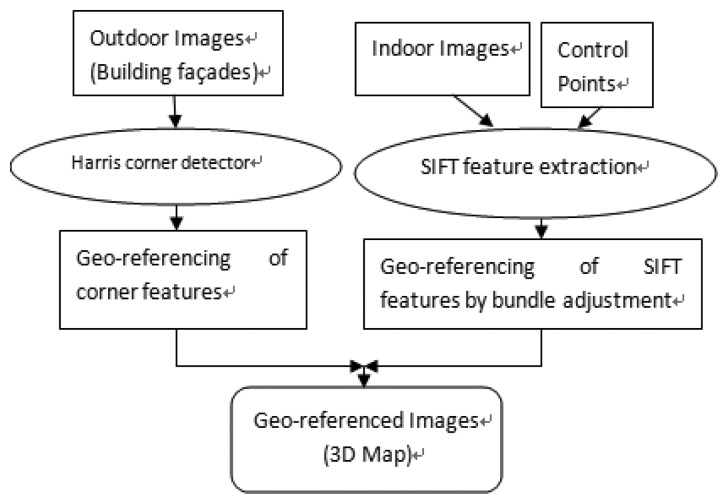
Flowchart for the mapping procedure.

To produce geo-referenced 3D maps for indoor positioning, ground control points have been set up and the images of the navigational environment are collected. Then SIFT feature points are extracted from these images and feature matching is performed between images with overlapped areas to produce tie points. These tie SIFT feature points are then geo-referenced through the least-square solution of photogrammetric bundle adjustment. The geometric accuracy of the map depends on the accuracy of geo-referencing. The overall flowchart for mapping is shown in Figure 4. At the current stage of the research, a local coordinate system is used for indoor environments. Using the geo-located façade information, the local coordinates can be transformed to the desired real world coordinate system.

### 2.2. Image-Based Positioning

During the navigation process, query images are taken wherever self-localization is needed. Although the method varies for indoors and outdoors, the processes for the two environments are based on the same principles: by performing the image matching between a real time query image and reference images, 3D coordinates are transferred through common features from reference images to the query image. The innovation is that these 3D features are used in the same way as ground control points are used. We give them the name pseudo ground control points (PGCP). By obtaining 3D points and their 2D positions on the query image, camera position and orientation of the query image can be determined through space resection.

Here we use the classical method for position resolution: space resection based on a least squares solution of linearised collinearity equations. This method is normally used to compute the exterior orientation of a single image. This procedure requires known coordinates of at least three object points which do not lie on a common straight line. The theory lies in that the bundle of rays through the perspective center from the reference points can fit the corresponding points in the image plane in only one unique (camera) position and orientation [22].

One of the major characters for a least squares based solution is that it needs an initial value for each unknown parameter to start the adjustment iteration. Therefore, the GPS data provides the initial positions and compass chip on mobile devices registers magnetism in three dimensions, which gives initial orientation values. By using the least square adjustment, user position and camera orientation in 6DoF can be achieved using vision measurements.

The least squares models are listed as Equations (1) and (2). It provides highly accurate results in the presence of redundant measurements:

(1) l+v=Ax

(2) $$D=\sigma_{0}^{2}Q$$

in which Equation (1) denotes the function model, Equation (2) the stochastic model and σ_0_ the a priori standard deviation of measurements.In Equation (1)***l*** denotes the observation, image measurement of reference points in this case; ***A*** denotes the design matrix; ***x*** denotes the unknowns, camera exterior orientation in this case; ***v*** denotes the residues. In Equation (2)***D*** denotes the variance covariance matrix of observations; σ_0_ denotes a priori standard deviation and ***Q*** the cofactor matrix. Using this model, the covariance matrix for the estimated camera external parameters (***C****_x_*) can be obtained using Equation (3), in which ***P*** represents the weight matrix. It is listed as:

(3) $$C_{x}=\sigma_{0}^{2}{\left(A^{T}PA\right)}^{-1}$$

Primarily the accuracy of camera external parameters is a function of point distribution and relative positions between the reference objects and the camera [23]. In our case, the relative positions change during navigation process, therefore the accuracy of positioning largely depends on the geometry of PGCPs. To evaluate the impact of geometry, the covariance of ***x*** will be simplified to:

(4) $${\text{C}}_{\text{x}}=\sigma_{0}^{2}{\left({\text{A}}^{\text{T}}\text{A}\right)}^{-1}$$

In fact, the elements in the trace of the matrix **(*A****^T^****A*)^−1^** are functions of the geometry only. In the GPS community, DOP values are used to represent the effect of satellite geometric distribution on the accuracy of a navigation solution. In our image-based positioning system, we give DOP values for resolved camera external parameters (position and orientation) to evaluate the precision, which is influenced by PGCP geometry. The diagonal of the matrix **(*A****^T^****A*)^−1^** is calculated as:

(5) $${\left(A^{T}A\right)}^{-1}=\left(\begin{array}{llllll} G_{x}^{2} & & & & & \\ & G_{y}^{2} & & & & \\ & & G_{z}^{2} & & & \\ & & & G_{\omega }^{2} & & \\ & & & & G_{\varphi }^{2} & \\ & & & & & G_{\kappa }^{2} \end{array}\right)$$

Then we give DOP values for 6DOF, which are calculated as follows:

(6) $$X\text{DOP}=\begin{array}{ccc} G_{x} & \text{YDOP}=G_{y} & \text{ZDOP}=G_{z} \end{array}$$

(7) $$\text{PDOP}=\sqrt{G_{x}^{2}+G_{y}^{2}+G_{z}^{2}}$$

(8) $$\omega \text{DOP}=\begin{array}{ccc} G_{\omega } & \varphi \text{DOP}=G_{\varphi } & \kappa \text{DOP}=G_{\kappa } \end{array}$$

(9) $$\text{ADOP}=\sqrt{G_{\omega }^{2}+G_{\varphi }^{2}+G_{\kappa }^{2}}$$

in which the PDOP represents the position DOP, while the ADOP represents Orientation DOP.

Therefore, the main contribution of the system methodology is the use of geo-referenced feature points as pseudo ground control points for positioning resolution; and major factor that influences positioning accuracy is the geometry (number& distribution) of these PGCPs. Greater details of the positioning solutions in outdoor and indoor environments are presented in the following two sections, respectively.

## 3. Outdoor Positioning

In urban canyons or indoor areas, GPS positioning accuracy can be degraded because the signal may suffer from blockage, multi-path effects, *etc.* For single point positioning (SPP) used on people's mobile devices, the accuracy can be 10 s of meters or worse. Therefore, image-based methods are used to mitigate the deficiency. However, if solely replacing GPS with image-based methods, retrieving images from a large image database that covers the whole navigation route will be time consuming and the computational load is not affordable for mobile devices. Therefore, we propose a multi-step solution: firstly we use GPS data to narrow down the search space; then a voting strategy is used to find reference images corresponding to the query view among the localized image space; finally, a hybrid technique is proposed that uses the measurements from GPS, digital compass and visual sensor onboard to calculate the final positioning result in 6DoF for outdoor environments. The overall outdoor positioning procedure is shown as follows:

- Step1: Take query image with GPS and compass measurements;
- Step2: Use GPS data to localize image space;
- Step3: Retrieve from the candidate image space the reference image(s) that contain the scene corresponding to the query image. If no correspondence is found, go to step 1 with enlarged search space. If yes, continue;
- Step4: Outdoor positioning resolution.

### 3.1. Using GPS to Localize Image Space

In the central database, we store the 3D maps in the form of reference images and their corresponding geo-information. We treat each building as a record and describe its contours with line segments which contain coordinates of both ends (corners). When a user is navigating (walking or driving) through the space, images are taken when position information is required.

**Figure 5.**
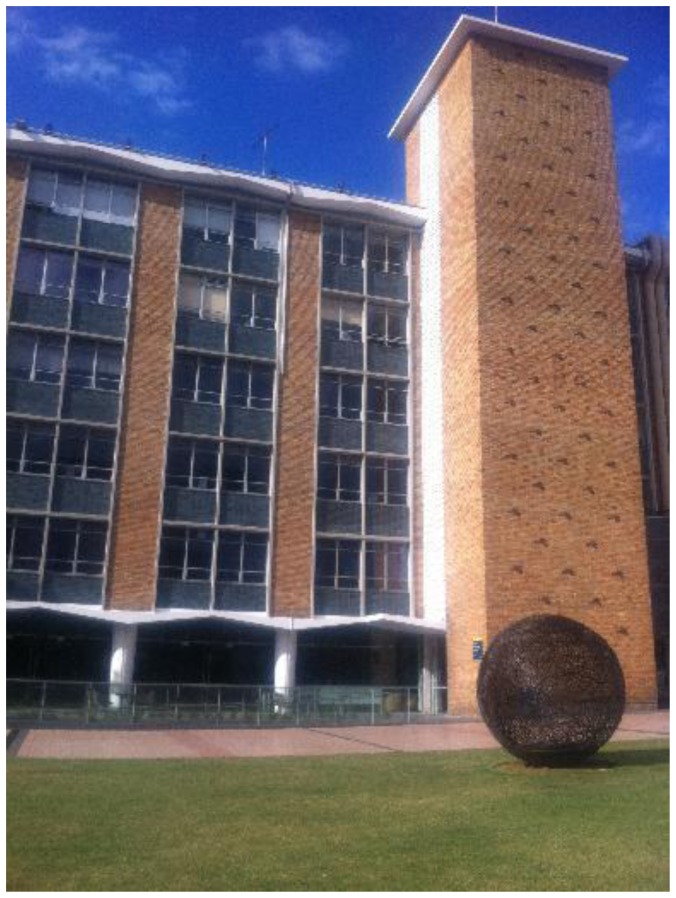
Query image No. 3 with GPS tag information: Zone 56, Latitude: −33°55′5.40120′, Longitude: 151°13′52.79880′, Altitude: 33.92 m; digital compass measurement: 34°NE.

Whenever a query image is taken with its GPS position tagged, the initial position is given by the GPS tag and the initial orientation is given by the digital compass onboard (e.g., P for query image No. 3 in Figure 5). A circle will be generated with the center at current GPS tagged position, and the radius (*r*) determined by a threshold (*n*), which is a certain magnitude of the horizontal precision of the GPS reading (σ). By default n = 1:

(10) r=n*σ

**Figure 6.**
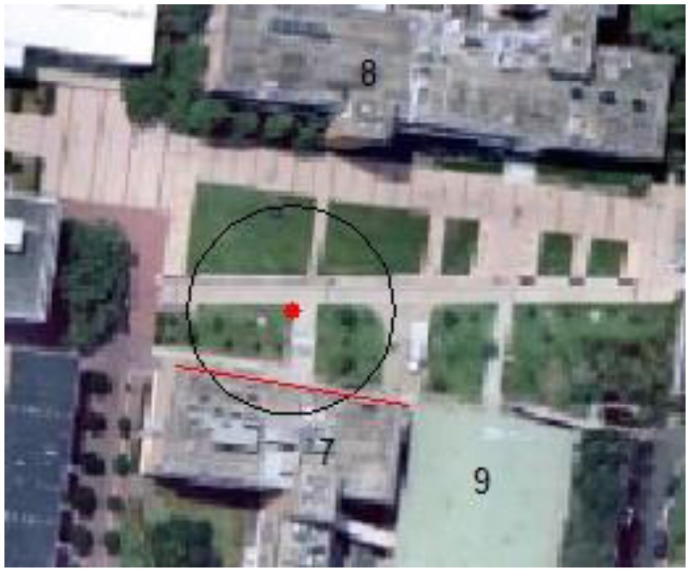
Given a GPS position data from mobile device at P shown with red dot, a circle is drawn with 20 m radius representing the search space. Line segment from building No. 7 crossed with the circle, so reference images of building No. 7 are chosen for image space.

**Figure 7.**
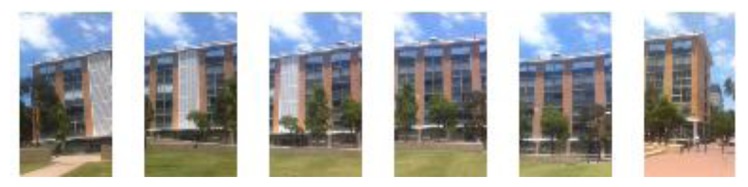
Image space created for P including façade images of Building No. 7.

Then the system will search for landmarks (corner points or line segments) appearing within the circle. The mobile device will load the images related to the landmarks that have been found. For buildings, the algorithm calculates the shortest distance between center point and the building line segments. If this distance is shorter than the radius, the segment line must cross with the circle. The corresponding building(s) images will be chosen and form an image space for further processing. Figure 6 illustrates the calculation process for user position P, and the resulting image space is shown in Figure 7. By using GPS information to narrow down the search space, the query image will need to match with a small image space rather than a whole image database at later stage.

### 3.2. Image Retrieval Using SIFT-Based Voting Strategy

The goal of this process is to retrieve, in the candidate image space, the reference images with the scene corresponding to the query image. If no corresponding image is found within current candidate image space, the procedure goes back to previous step and enlarges the threshold (*n*) to recalculate the crossed landmarks. And the images in previous image space are removed and new image space is produced. In the example of query image No. 3 at point P, image space in Figure 7 is removed because no corresponding image has been found, and a new image space is generated after the search space has been enlarged.

**Figure 8.**
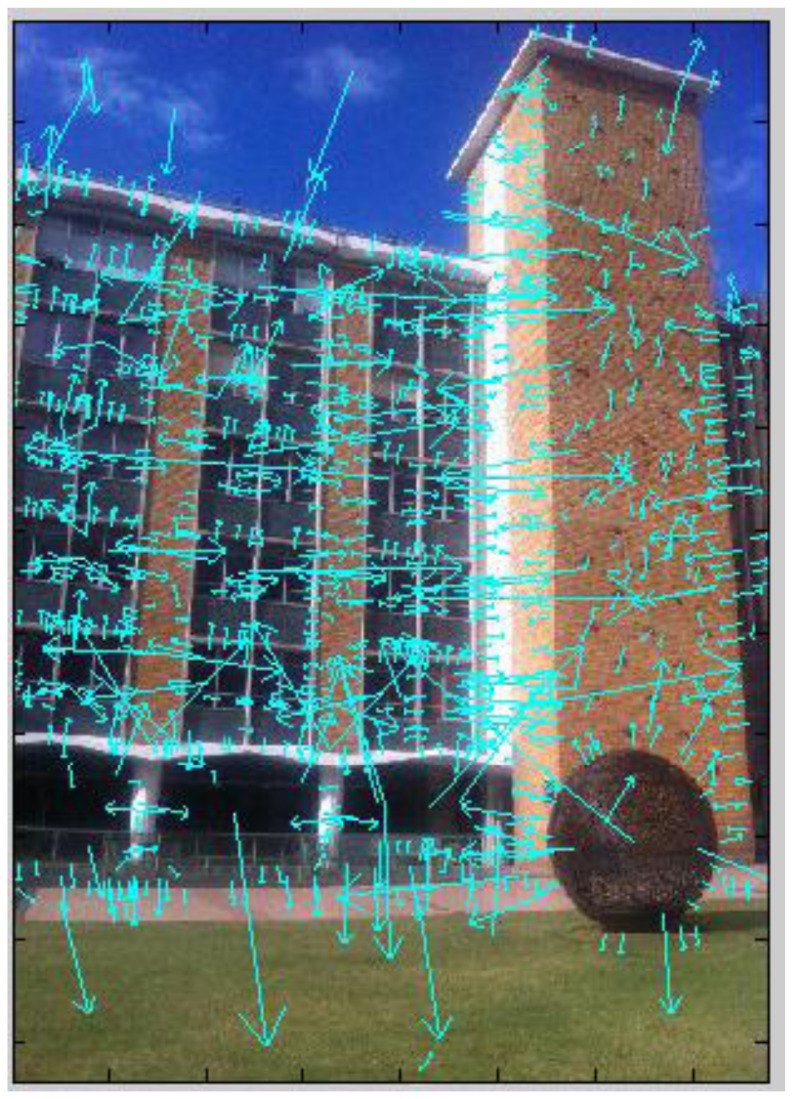
SIFT features extracted from the query image No. 3.

**Figure 9.**
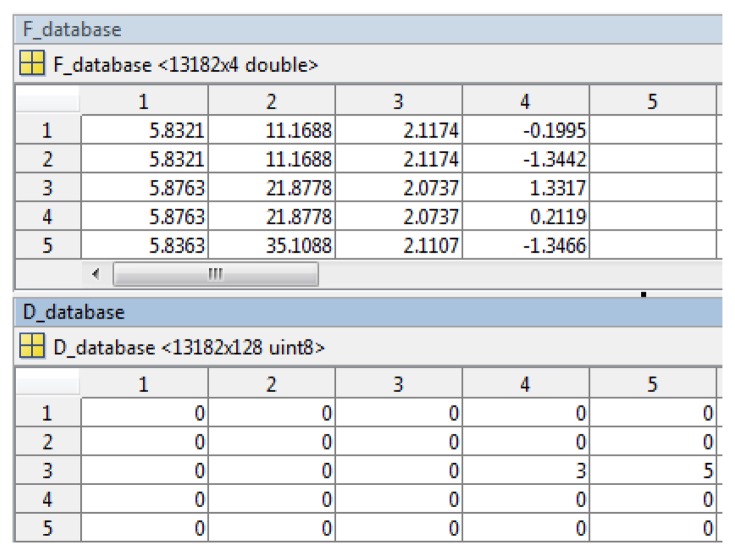
Reference feature database generated for the candidate image space: 13182 features from 20 reference images in the image space.

In the system this process identifies the target building and prepares the corresponding reference images for outdoor and indoor navigation. We first use a voting strategy to find candidate reference images, then check the geometric consistency to detect mismatches and remove mismatched reference images from the image space. First, the SIFT features are extracted for the query image (e.g., Figure 8). Then a reference feature database is generated for the candidate image space. SIFT feature points are extracted from each candidate reference image and put into two sub datasets: keypoint database (F_database) and descriptor database (D_database). F_database contains SIFT keypoints' location, scale, orientation using four parameters, and the D_database contains 128 dimensional SIFT descriptors. Figure 9 gives the visualization of the feature database generated from the image space.

**Figure 10.**
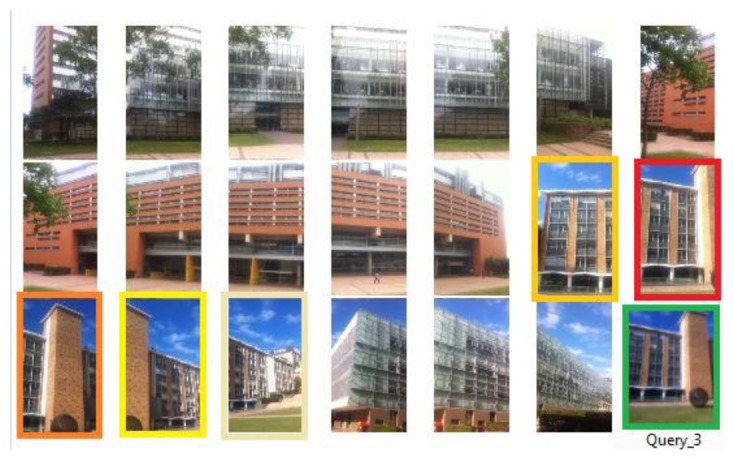
Image retrieval: 5 top ranked images have been identified from the image space with 20 reference images. Query image has a green border when the five top voted reference images have borders from dark red to light yellow, the darker the colour the higher rank it has (which indicates greater relevance). All top voted reference images indicates the same target building, BD No. 8.

Secondly, SIFT matching is performed between the query image and the newly generated reference feature database (Figure 10) to find corresponding reference images. A K-NN (here k = 3) search function is used to find the k nearest neighbours from the feature database for the feature points in the query image. Each correspondence adds one vote to the reference image it belongs to. The reference images with greater numbers of votes obviously have higher chance of containing common scene with query image. Therefore, by ranking in descending order of the number of votes, the top *m* (5 in our case) reference images are chosen as ones corresponding to the query scene and retrieved from the candidate image space. Specific building(s) that covered by the query view can also be identified.

**Figure 11.**
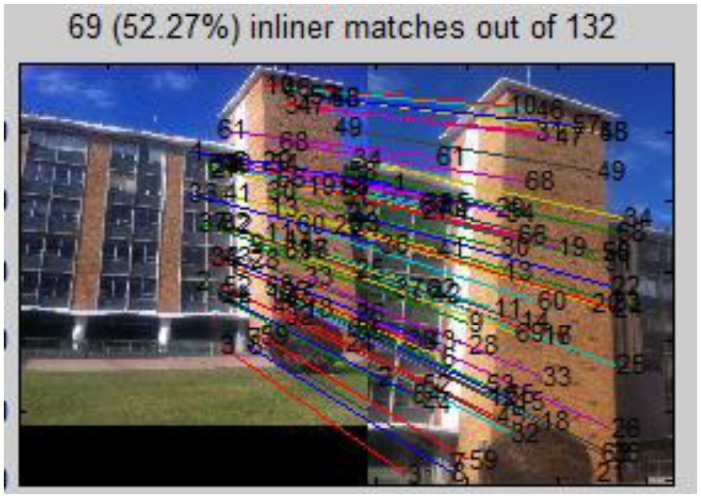
SIFT based matching between query image and the 2nd ranked reference image No. 15 with mismatches removed by RANSAC; Average correlation coefficient score ρ̄ = 0.84.

**Figure 12.**
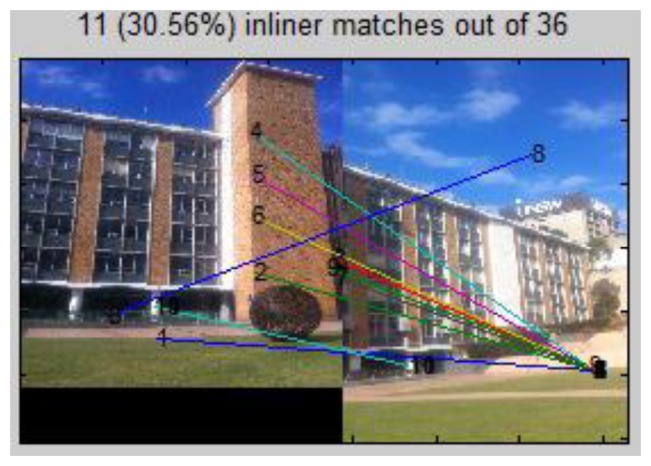
SIFT based matching between query image and 5th ranked reference image No. 17; Average correlation coefficient score ρ̄ = −0.10.

To improve the robustness of the system, a further step is to check to the correctness of the top voted images based on pair-wise geometric consistency. This process can detect any falsely ranked/selected reference images as well as remove mismatches. First RANSAC is used to estimate the homography (projective transformation) between the two images, and remove mismatches (Figure 11). A new method we proposed in previous research [24] that utilizes cross-correlation information to check the quality of the homography model built by RANSAC is used here to further ensure the correctness of matching. We calculate the cross-correlation (Equation (11)) for each pair of inliers, in which G_uv_ and $G_{\text{uv}}^{\prime }$ represent the intensity values of the two correlation windows, respectively, whereas μ(G) and μ(G′) denote their average intensity. In Equation (11) ρ(G, G′) varies from −1 to 1, the closer to 1 the higher correlation, and here indicates the bigger similarity between two patches and greater possibility to be correct corresponding points. Therefore we calculate the cross-correlation for each pair of reliable matches (inliers) for every single matching process. An average correlation ρ̄ is calculated for all the matched (reliable) points produced by one matching (one H is generated). If ρ̄ is close to 1, the estimated homography model H is very accurate and the two images are a correctly matched pair; the reverse would also apply. The threshold for ρ̄ is set to 0.75 in the system. In Figure 11 the score is higher than the threshold, which indicates it is a correct matched pair; while in Figure 12 the score shows the inverse situation. It can be easily observed that reference image No. 17 indeed shares no common view with the query image, and it will be deleted from the reference list. If all the reference images in the current image space have been deleted, the procedure goes back to previous step (3.1) to recalculate. Therefore in this way the candidate image space is further filtered, so that it contains only the reference images with corresponding views in the query image. It narrows down reference images to four in the example:

(11) $$\rho \left(G,G^{\prime }\right)=\frac{\sum_{u=-w}^{w}\sum_{v=-w}^{w}\left(G_{uv}-\mu \left(G\right)\right)\left(G_{uv}^{\prime }-\mu \left(G^{\prime }\right)\right)}{\sqrt{\sum_{u=-w}^{w}\sum_{v=-w}^{w}{\left(G_{uv}-\mu \left(G\right)\right)}^{2}.\sum_{u=-w}^{w}\sum_{v=-w}^{w}{\left({G^{\prime }}_{uv}-\mu \left(G^{\prime }\right)\right)}^{2}}}$$

### 3.3. Outdoor Positioning

In this section, we introduce a hybrid technique that uses the GPS as well as the digital compass measurements, and image-based positioning technology for outdoor positioning. In fact it deals specifically with urban environments with artificial landmarks.

**Figure 13.**
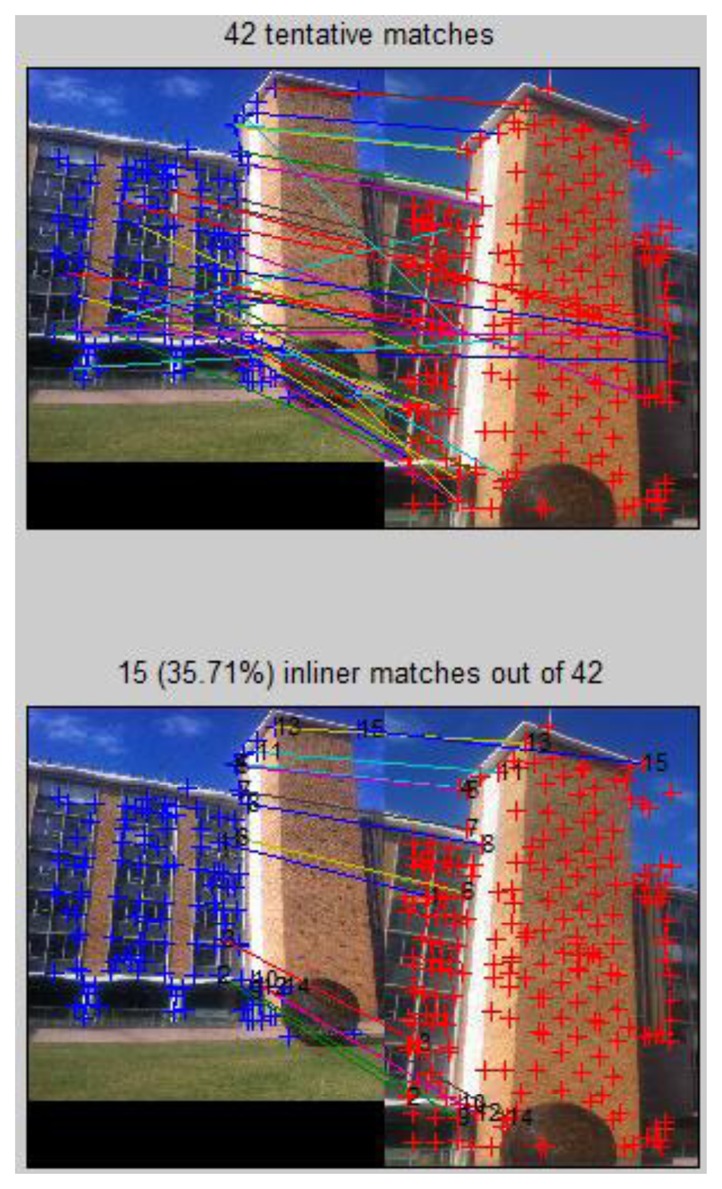
Query image matching with reference image No. 15 using Harris/SIFT method; Harris corner features are tagged by blue and red crosses respectively, and matched corner features using SIFT descriptor matching are shown by lines.

**Figure 14.**
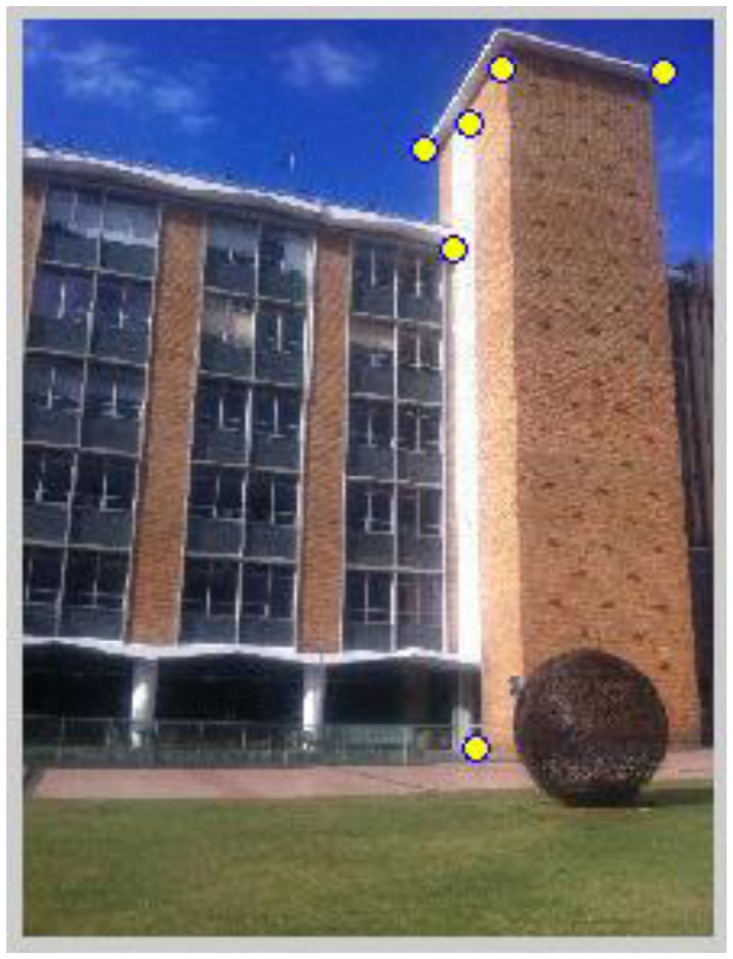
PGCPs generated for the query image No. 3, which are shown with yellow dots.

After reference images have been identified in the previous step, positioning is carried out based on the matching between the query image and identified geo-referenced images. Since the outdoor reference images are geo-referenced through the corner features, to ensure corners to be matched in the query image as well as to strengthen the robustness of matching, we apply a combined use of the Harris corner detector and the SIFT descriptor (referred by Harris/SIFT method). Firstly, the Harris corner detector is used to extract corner features from the query image and SIFT descriptors are computed at the positions detected by Harris detector on the query image. In the meantime, SIFT descriptors are also generated for geo-located corner features on the reference image that to be matched. Then feature matching is carried out between the two images based on SIFT descriptor matching. RANSAC is used to remove mismatches. As shown in Figure 13, 15 pairs of correct matches are found, among which four are geo-located corner points (No. 4, 8, 13, 15) that are identified in Figure 1 and Table 1. Therefore, the 3D geo-locations of these points are transferred from reference image No. 15 to the query image, which can be then used as PGCPs for positioning resolution. More PGCPs can be generated by matching the query image with all the corresponding reference images selected by previous step. The given query image obtained 6 PGCPs as illustrated in Figure 14.

**Figure 15.**
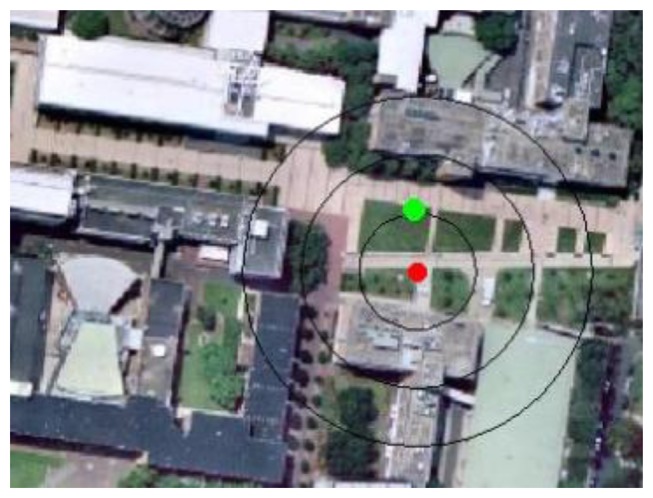
Positioning result for the query image shown with green dot. The red dot indicates the location determined by the GPS, with the black circles show the process to enlarge the search space (1–3 times of its horizontal precision).

After enough PGCPs have been generated, the methodology introduced in Section 2.2 is used to resolve the user position and orientation of the query image. Although space resection based on a least squares solution can provide a relatively accurate result, it requires a good initial value for the least squares adjustment to converge. This is where the raw GPS and digital compass measurements come into play. Normally standalone GPS or AGPS is used on mobile devices, which can only provide either low accurate positioning or unstable performance in dense urban area. Therefore, the GPS provides the initial positions and compass chip on mobile devices registers magnetism in three dimensions, which gives initial orientation values. By using image-based positioning, user position and camera orientation in 6DoF can be achieved. For the query image, the computed result is shown in Figure 15.

## 4. Indoor Positioning

**Figure 16.**
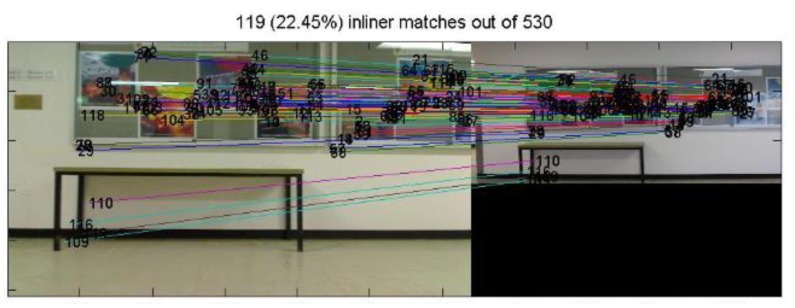
Matching between real time query image No. 64 and geo-referenced map image.

**Figure 17.**
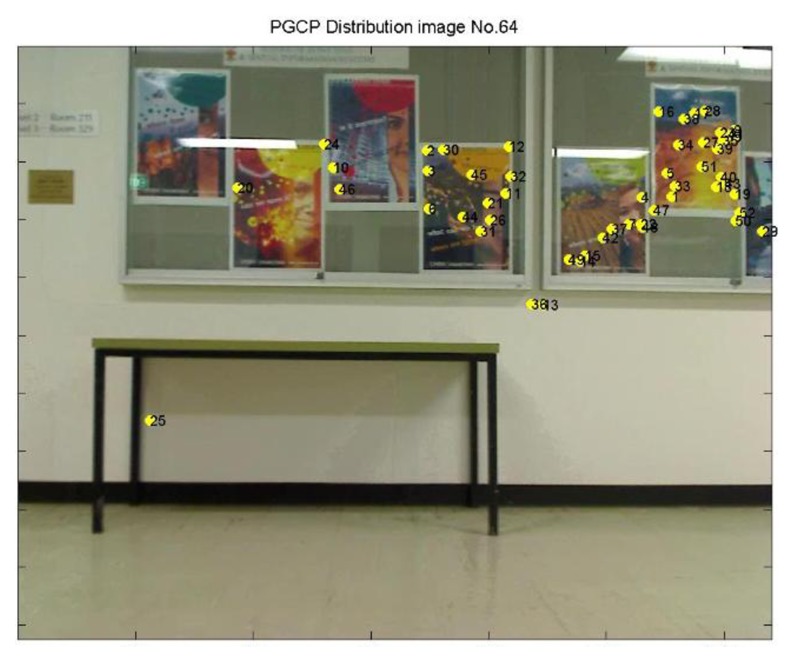
PGCPs (yellow dots) on real time query image No. 64.

Since the target building has been identified in Section 3.3, when a user walks into the building, the geo-referenced images of its indoor environment are loaded. Then real time images are taken, another image matching based on SIFT is carried out between the real-time query image and the geo-referenced images for position resolution (e.g., Figure 16). The RANSAC approach and the cross correlation information are used to ensure the correctness of matching. When any of the SIFT feature points from the geo-referenced image(s) are found to correspond with the ones on the query image, the geo-information it carried can be transferred to its counterpart. Therefore, matched SIFT features on the query image obtain both image coordinates from matching process and 3D coordinates from the geo-referenced images. These coordinates can later define the positions of pseudo ground control points (PGCPs) for space resection based positioning in the final stage (e.g., Figure 17).

However, one major difference between outdoor positioning and indoor positioning is that the 3D object space coordinates of PGCPs are photogrammetrically determined, which are not accurate enough to be used as error-free reference or held fixed. Therefore they are introduced into the system as observed unknowns (pseudo observations) with a corresponding weight. We introduced modified space resection for indoor positioning resolution. The Gauss-Markov Model for with stochastic constraints is as follows:

(12) $$\begin{array}{cc} A_{t}+BX-l_{1}=v_{1}, & \;l_{1}\sim \left(0,\sigma_{0}^{2}{P_{1}}^{-1}\right) \end{array}$$

(13) $$\begin{array}{cc} IX-l_{2}=v_{2}, & \;l_{2}\sim \left(0,\sigma_{0}^{2}{P_{2}}^{-1}\right) \end{array}$$

Combine Equations (12) with (13):

(14) $$\begin{array}{cc} \left[\begin{array}{cc} A & B\\ 0 & I \end{array}\right]\;\left[\begin{array}{c} t\\ X \end{array}\right]-\left[\begin{array}{c} l_{1}\\ l_{2} \end{array}\right]=\left[\begin{array}{c} v_{1}\\ v_{2} \end{array}\right], & \;\left(\begin{array}{cc} P_{1} & 0\\ 0 & P_{2} \end{array}\right) \end{array}$$

in which ***A*** is a matrix containing partial derivatives with respect to the exterior orientation parameters; ***B*** with respect to the three coordinates of the PGCPs; ***t*** contains the incremental changes to the initial values of external orientation parameters; and ***X*** contains the incremental changes to the initial values of ground coordinates of PGCP; ***l*_1_** denotes the observation, image measurement of reference points in this case, ***P*_1_** is its corresponding weight; ***l*_2_** denotes the pseudo observation, object space coordinates of PGCPs in this case, ***P*_2_** is its corresponding weight; ***v*_1_** and ***v*_2_** denotes the residues. From Equation (14) we deduce that:

(15) $$Q_{tt}={\left(A^{T}P_{1}A-A^{T}P_{1}B{\left(B^{T}P_{1}B+P_{2}\right)}^{-1}B^{T}P_{1}A\right)}^{-1}$$

From Equation (15) we can clearly observe that the final positioning precision for indoors using a modified space resection model is affected by two major elements: ***A*** and ***B***, which determined geometry (Equation (4)), and the accuracy of measurements: image measurement (***P*_1_^−1^**) and 3D object space coordinates of Pseudo Ground Control Points (***P*_2_**^−1^). The stochastic model is built based two main factors: SIFT feature extraction accuracy, and the geo-referencing accuracy during the 3D mapping process.

## 5. Experiments

In the experiment, a seamless positioning and navigation test from outdoors to indoors was carried out. We chose a path on the UNSW campus and entered a building then went back to the path. It has seven pre-surveyed GPS ground control points (GCPs) on the way, as well as buildings on both sides, which can simulate the situation of urban area. Images of the building facades as well as indoor environment (test scene) were recorded in the database and geo-referenced 3D maps were generated for positioning. Then a user walked along the path and entered a nearby building. The position of each epoch when images were taken and the trajectory are resolved based on the image-based system developed. The data is post-processed using Matlab 2011a and an orthophoto of the UNSW campus.

### 5.1. Outdoor Navigation and Analysis

During the outdoor test, a user holding a mobile handset walked along the path and took (query) images for self-localisation. The device for experiment was an iPhone 4 smart mobile phone, which integrates a backside-illuminated 5 megapixel rear-facing camera with a 3.85 mm f/2.8 lens, and employ an assisted GPS system (A-GPS) and a specialized integrated circuit chip as the iPhone's digital compass for navigation. The ‘user’ passed by each of the 7 GPS GCPs and took images of the environments on the seven sites, and randomly took another 11 images along the path. Totally 18 epochs were resolved.

Firstly, the performance of outdoor image-based positioning with its accuracy was investigated by calculating the user positions and orientations at the 7 GPS GCPs through the proposed method and compared them with surveyed true values. The accuracy of image-based method and standalone GPS is also compared. The 6DoF results are shown in Table 2 and the accuracy revealed by RMSE in Table 3. It can be seen that the GPS measurements in the urban environment are poor, around 20 m in our experiments. By using the proposed method, the accuracy has been improved to around 10 m revealed in the test.

**Figure 18.**
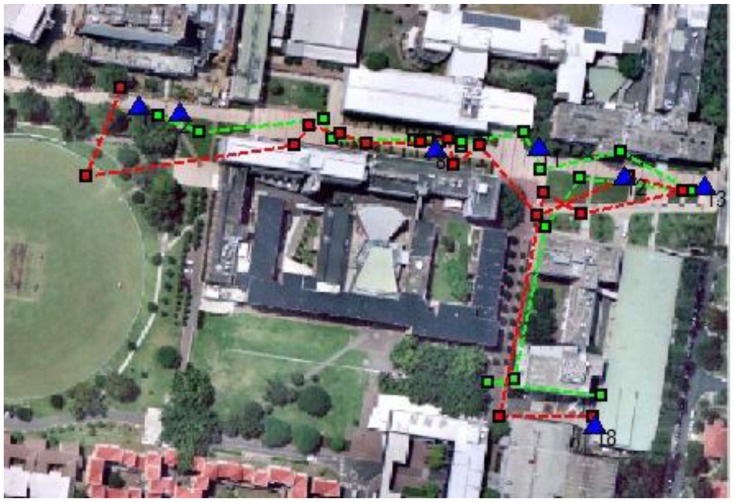
Red dash line shows the trajectory obtained from the build-in GPS receiver, while green dash line shows the calculated results; blue triangles represent the true GCPs that user passed by.

**Figure 19.**
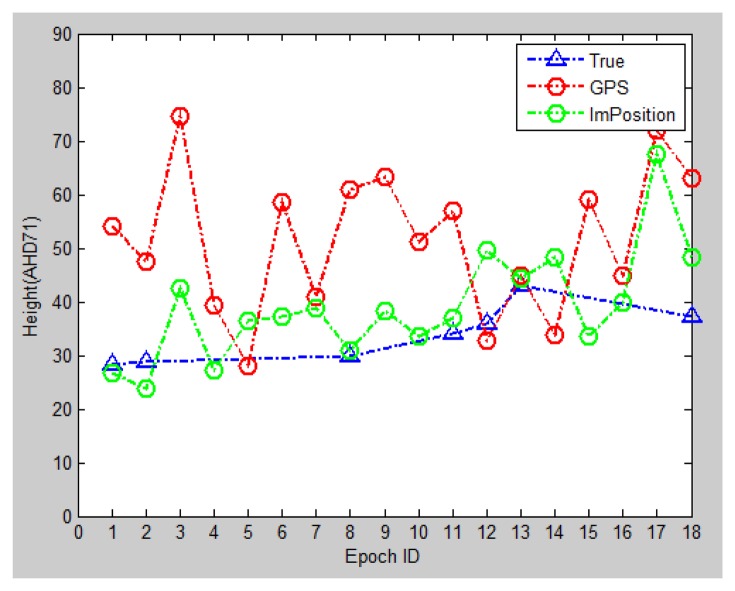
Height: blue icons represent true values; red ones are altitude measured by the device; green ones indicate the calculated results.

Secondly, the overall trajectory, including 18 epochs, is calculated and shown in Figure 18 (horizontal) and Figure 19 (vertical). It can be easily observed that horizontally the GPS measurements present a few jumps (e.g., epoch No. 2) and intersected track, which are not true; while the image-based method provides a trajectory closer to the true trajectory. Meanwhile, the height information provided from GPS deviate largely from true values, while the system results are much improved.

**Figure 20.**
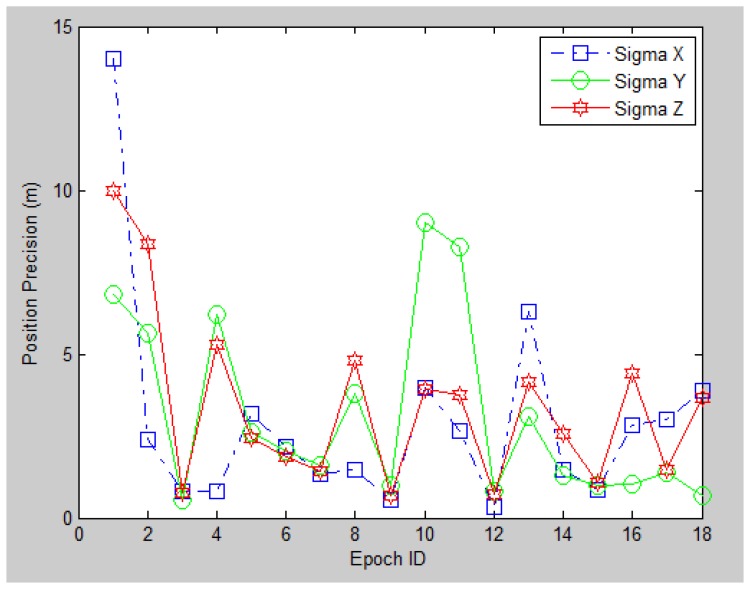
Position precision for the 18 epochs.

**Figure 21.**
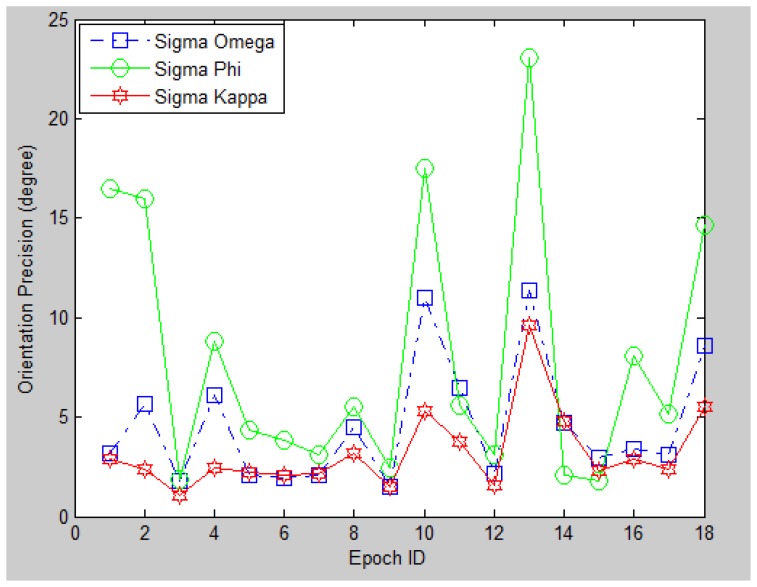
Orientation precision for the 18 epochs.

Thirdly, we investigate the theoretical precision for the image-based position resolution in 6DoF by using their estimated standard deviation, as shown in Figures 20 and 21. It can be observed that most of the epochs have a 0–5 m standard deviation on each direction, while the orientation standard deviation mostly between 0–10 degrees. The theoretical precision is consistent with the accuracy evaluated by the seven check points. Moreover, it is noticed that certain epochs have very low precision (large standard deviation) compared with other epochs (e.g., Epoch No. 1, 2, 10, 13). The reason behind is poor geometric distribution of PGCPs on query images. Such a phenomenon has also been found and further investigated in the indoor experiment. The main contributing factors that determine the PGCPs geometry are the geo-referenced 3D feature density of the reference images, the quality of image matching and most importantly the covered scene of the query image. Therefore one possible way to improve the outdoor positioning performance is to include greater number of corner features with better distribution when taking the query image. In other words, such image-based method performs the best in areas where artificial landmarks are sufficient (like deep urban canyons), which is a complementary character for satellite-based navigation system.

In conclusion, for SPP used on people's mobile devices in urban canyons, the accuracy can be 10 s meters or worse, and varies significantly depending on the signal. Image-based methods, on the contrary, can provide stable results with relatively better accuracy as long as enough visual features are covered by the query image.

### 5.2. Indoor Navigation and Analysis

The outdoor navigation passed by Building No. 8, and related indoor reference images were loaded. Image-based positioning was carried out in the mapped indoor area of Building No. 8. The motivation of the paper is to design a system that can be easily applied to mobile platforms such as smartphones and moving vehicles. Besides, in the limited space of the indoor environment, we need to evaluate the performance based on a clear trajectory, which means higher sampling rate is required. Therefore in the second experiment, a calibrated video camera (Logitech Webcam Pro2000) was mounted on a moving vehicle with sampling rate of 1 Hz. Its relative position to the vehicle was fixed, which means the experiment was partially controlled: camera height Z:−0.725 m. We did the positioning by extracting image frames from the video and match with the geo-referenced images frame by frame. Each frame is an epoch; a position in 6 degree of freedom (6DOF) was calculated. This experiment aims at evaluating the indoor positioning accuracy as well as the main factors that determine positioning accuracy of the system in general.

**Figure 22.**
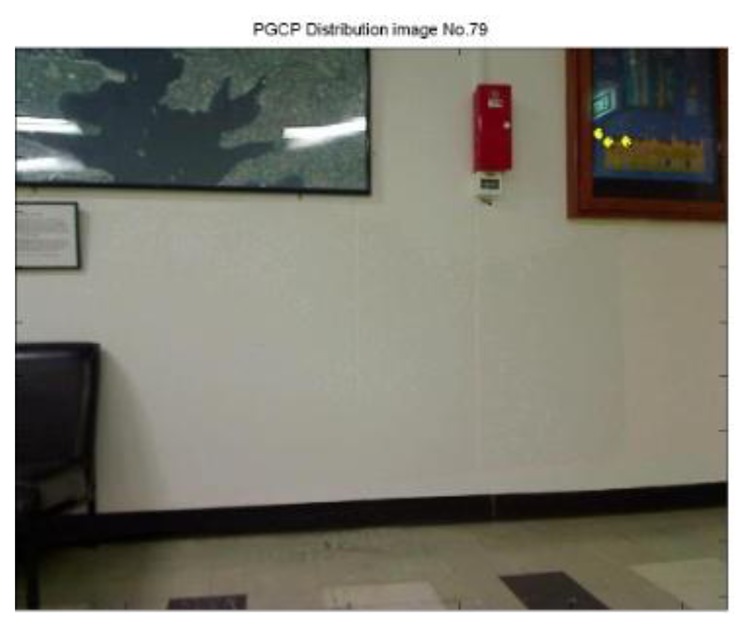
PGCPs on epoch No. 79, which failed to give a position result.

**Figure 23.**
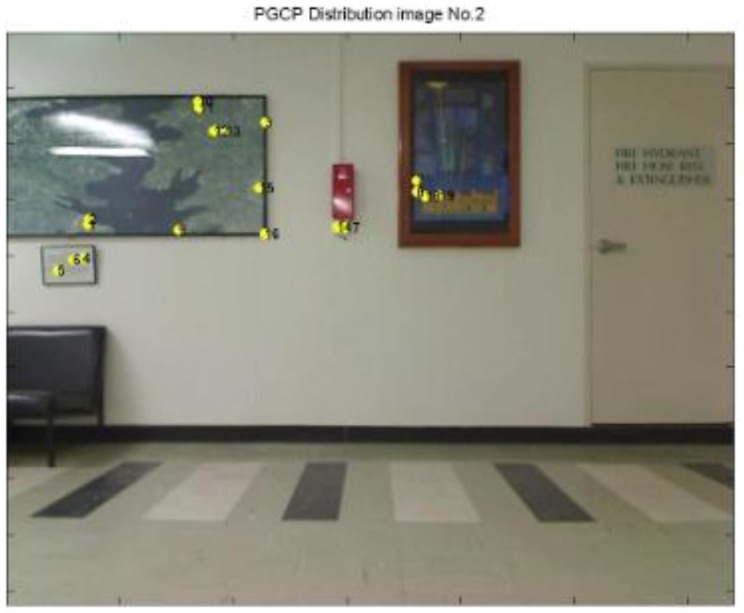
PGCPs on epoch No. 2, which gave a position result.

From the video, a total 83 epochs (frames) were generated and calculated, 20 epochs failed to determine the camera position, which is failure rate at 24.1%. It is observed that the failed results all come from insufficient PGCPs (e.g., Figure 22), and a normal resolution need more PGCPs that are evenly distributed in the scene (e.g., Figure 23). It is noted that with greater number of PGCPs are required for indoor positioning than that of outdoor because of the modification of function model.

**Figure 24.**
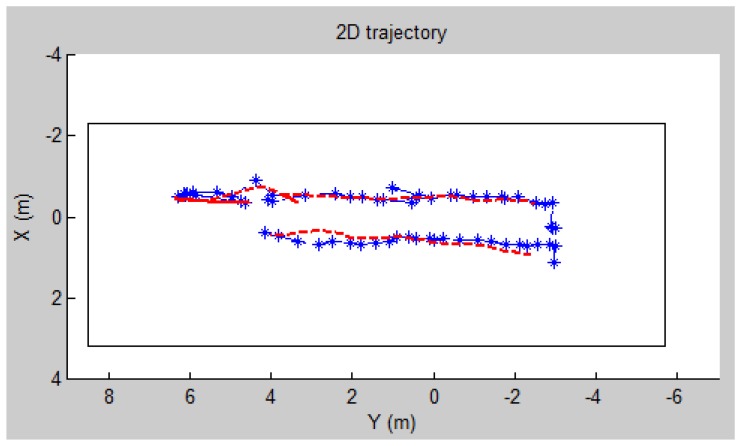
Two dimensional trajectory of indoor navigation recovered by the image-based system (blue line) with reference to the Photomodeller results (red line).

**Figure 25.**
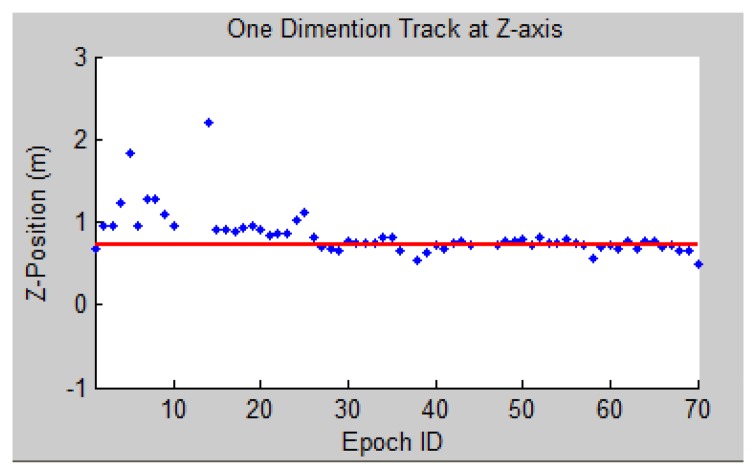
Z positions of indoor navigation calculated by the system (blue dots) with reference to the controlled value (red line).

As GPS GCPs are not available in indoor areas, we use commercial software Photomodeller to determine camera positions and then use them as references to evaluate the system produced results. Within 10 m distance, the software can normally achieve centimeter level accuracy. From Photomodeller 60 reference epochs were generated and the two systems have 55 epochs in common. The trajectory of indoor navigation is shown in Figure 24 (horizontal) and Figure 25 (vertical). The RMSE of the calculated positions is shown in Table 4.

**Figure 26.**
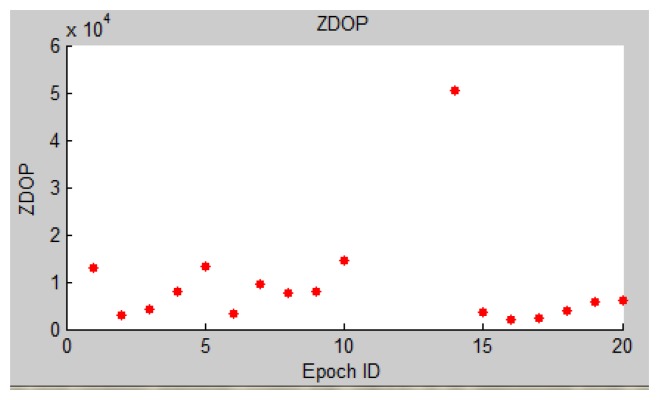
ZDOP of epoch 1–20.

It can be observed that the accuracy of indoor positioning is round 20 cm level. From Figure 25 it is easy to discover that the positioning accuracy in Z varies largely on the first few epochs, so we hope to find the reason behind the accuracy fluctuation. From Equations (4) and (15), it is identified that the major factor that influences the positioning accuracy is the PGCP geometry. To test the correctness of the theory, ZDOP (Equation (6)) of epochs 1–20 is calculated and shown in Figure 26. By comparing Figure 26 with Figure 25, it can be discovered that big DOP values, which means low precision, is the main contributing factor to the inaccurate results (e.g., epochs No. 5 and 14). And unevenly distributed texture leads to such an occasion. A possible solution is to set up artificial marks in areas where no texture can be found, such as blank walls.

## 6. Conclusions

In this paper, we have presented a comprehensive system that adopts a hybrid image-based method with combined use of onboard sensors (GPS, camera and digital compass) to achieve a seamless positioning solution for both indoor and outdoor environments. The main contribution of this paper is the use of geo-referenced images as 3D maps for image-based positioning, and the adoption of multiple sensors to assist the position resolution. Various image matching methods are used for different scenarios. Experiments have demonstrated that such a system can largely improve the position accuracy for areas where GPS signal is degraded (such as in urban canyons). The system also provides excellent position accuracy (20 cm) for indoor environments.

The nature of such system has also been studied. The final position accuracy is mainly determined by the geometry (number and distribution) of the identified geo-referenced features (PGCPs). Therefore, the geo-referenced 3D feature density of the reference images, the quality of image matching and most importantly the covered scene of the query image become the essential elements of the solution. The paper also reveals the major challenge for such system, that is, it largely depends on the texture of the view. For outdoor environments, the shortage of texture because of poor lighting conditions may pose tremendous challenges to the system. In the case of changed landscape, which is more likely for indoor environments, such as change of posters or movement of furniture, such an approach will suffer from incorrect results due to mismatches. It is noted that for indoor positioning, another limitation is that a GPS signal is not available. The current approach is to use the previous GPS data to identify the building and load all the map images of the interior of the building for indoor navigation. For further investigation, we intend to incorporate WiFi signal and used it in the same way GPS has been used: provide rough location to help narrow down the search space of map images and initial value for space resection. Besides, the use of WiFi can also reduce the chance of misidentified locations. Future work will focus on such aspects.

We believe that the system has potential to overcome a deficiency of the satellite based solution, since it targets GPS-challenged environments and works especially well for places with buildings/artificial landmarks and indoors. The required hardware, a single camera integrated with a GPS receiver and digital compass, can be easily found on people's mobile devices (smart phones, *etc.*). With the boom in LBS and growing attention to geo-spatial techniques for everyday life (e.g., GPS-tagged image), we hope such technologies can bring vision-based techniques for position and navigation to a new level and finally achieve ubiquitous positioning.

## Figures and Tables

**Table 1.** Geo-located corner points for reference image No. 15, X, Y in pixel and Easting, Northing and Height in meters.

**RefIM**	**PointID**	**X(pixel)**	**Y(pixel)**	**Easting(m)**	**Northing(m)**	**Height(m)**
15	807	83	108	336512.9	6245551	63.9
15	811	106	184	336511.4	6245548	56.9
15	806	154	49	336511.9	6245543	63.9
15	805	280	76	336519.8	6245542	63.9

**Table 2.** System calculated positioning results in 6DoF for the 7 GCPs.

**Epoch ID**	**Easting(m)**	**Northing(m)**	**Height(m)**	**Omega (degree)**	**Phi (degree)**	**Kappa (degree)**
1	336269.08	6245563.38	26.67	−89.30	1.44	71.35
2	336291.39	6245554.18	23.78	−119.22	−1.25	96.66
8	336435.40	6245546.05	30.97	−123.64	−5.74	117.53
11	336478.50	6245533.39	36.99	−103.93	−159.27	−64.17
12	336522.79	6245543.21	49.73	−139.19	173.80	−136.67
13	336562.16	6245522.26	44.43	−92.81	−12.54	−80.99
18	336511.91	6245409.58	48.33	58.16	153.63	12.12

**Table 3.** RMSE of GPS measurements and system calculated positions using surveyed values as true values.

**RMSE**	**Easting(m)**	**Northing(m)**	**Height(m)**
GPS measurements	20.37	19.59	21.00
Calculated	8.43	10.31	7.20

**Table 4.** RMSE for indoor positioning.

**RMSE**	**X(m)**	**Y(m)**	**Z(m)**
Calculated	0.126	0.281	0.137
